# Type of mRNA COVID-19 vaccine and immunomodulatory treatment influence humoral immunogenicity in patients with inflammatory rheumatic diseases

**DOI:** 10.3389/fimmu.2022.1016927

**Published:** 2022-10-13

**Authors:** Catherine E. Raptis, Christoph T. Berger, Adrian Ciurea, Diego O. Andrey, Christos Polysopoulos, Pierre Lescuyer, Tanja Maletic, Myriam Riek, Almut Scherer, Isabell von Loga, Judith Safford, Kim Lauper, Burkhard Möller, Nicolas Vuilleumier, Axel Finckh, Andrea Rubbert-Roth

**Affiliations:** ^1^ SCQM Foundation (Swiss Clinical Quality Management in Rheumatic Diseases), Zurich, Switzerland; ^2^ University Center for Immunology and Immunization Clinic, University Hospital Basel, Basel, Switzerland; ^3^ Translational Immunology, Department of Biomedicine, University of Basel, Basel, Switzerland; ^4^ Department of Rheumatology, Zurich University Hospital, University of Zurich, Zurich, Switzerland; ^5^ Laboratory Medicine Division, Geneva University Hospitals, Geneva, Switzerland; ^6^ Faculty of Medicine, University of Geneva, Geneva, Switzerland; ^7^ RheumaCura Foundation, Bern, Switzerland; ^8^ Division of Rheumatology, Geneva University Hospitals, Geneva, Switzerland; ^9^ Division of Rheumatology and Immunology, Inselspital, Bern University Hospital, Bern, Switzerland; ^10^ Division of Rheumatology and Immunology, St. Gallen Cantonal Hospital, St. Gallen, Switzerland

**Keywords:** SARS-CoV-2, vaccination, mRNA-1273, BNT162b2, anti-spike-IgG, waning immunity, rheumatic disease, immunosuppression

## Abstract

Patients with inflammatory rheumatic diseases (IRD) are at increased risk for worse COVID-19 outcomes. Identifying whether mRNA vaccines differ in immunogenicity and examining the effects of immunomodulatory treatments may support COVID-19 vaccination strategies. We aimed to conduct a long-term, model-based comparison of the humoral immunogenicity following BNT162b2 and mRNA-1273 vaccination in a cohort of IRD patients. Patients from the Swiss IRD cohort (SCQM), who assented to mRNA COVID-19 vaccination were recruited between 3/2021-9/2021. Blood samples at baseline, 4, 12, and 24 weeks post second vaccine dose were tested for anti-SARS-CoV-2 spike IgG (anti-S1). We examined differences in antibody levels depending on the vaccine and treatment at baseline while adjusting for age, disease, and past SARS-CoV-2 infection. 565 IRD patients provided eligible samples. Among monotherapies, rituximab, abatacept, JAKi, and TNFi had the highest odds of reduced anti-S1 responses compared to no medication. Patients on specific combination therapies showed significantly lower antibody responses than those on monotherapy. Irrespective of the disease, treatment, and past SARS-CoV-2 infection, the odds of higher antibody levels at 4, 12, and 24 weeks post second vaccine dose were, respectively, 3.4, 3.8, and 3.8 times higher with mRNA-1273 versus BNT162b2 (p < 0.0001). With every year of age, the odds ratio of higher peak humoral immunogenicity following mRNA-1273 versus BNT162b2 increased by 5% (p < 0.001), indicating a particular benefit for elderly patients. Our results suggest that in IRD patients, two-dose vaccination with mRNA-1273 versus BNT162b2 results in higher anti-S1 levels, even more so in elderly patients.

## Introduction

Patients with inflammatory rheumatic diseases (IRD) requiring immunomodulatory therapies represent a vulnerable population during the COVID-19 pandemic and may have an increased risk of poor COVID-19 outcomes (1, 2). Two mRNA COVID-19 vaccines, BNT162b2 (Comirnaty, Pfizer-BioNTech) and mRNA-1273 (Spikevax, Moderna), are currently available and have proven to be highly effective in preventing severe COVID-19 disease, including hospitalizations and deaths (3). However, patients on specific immunomodulatory treatments mount an attenuated antibody response following mRNA COVID-19 vaccination compared to healthy individuals and may be less protected (4–9). Data on whether the risk of breakthrough infections is increased as the immune response wanes over time and the impact of certain immunomodulatory medication on the level of antibodies in patients with different diseases are still under discussion (10–12).

The efficacy of therapeutic and prophylactic antibodies against the spike protein further supports the importance of a robust humoral immune response (13, 14). Vaccine-induced immune responses in immunocompromized individuals may, among other factors, depend on the type of vaccine received. The available mRNA vaccines both encode for the SARS-CoV2 spike protein but contain different amounts of mRNA. Moreover, the mRNA incorporates distinct proprietary nucleotide and sequence modifications to stabilise the mRNA and modulate its immune activation profile (15). There is evidence that these differences may be clinically relevant, as, compared to BNT162b2, vaccination with mRNA-1273 resulted in significantly lower infection and hospitalization rates in non-immunocompromized adults and US veterans and higher antibody levels in healthcare workers (16–18). To our knowledge, relevant studies comparing the vaccine-induced immune responses following a two-dose regimen of the mRNA COVID-19 vaccines in patients with rheumatic diseases mostly involved a single sampling timepoint, or have reported results in terms of the proportion of patients achieving seroconversion or passing a predefined threshold (5, 19, 20). However, since they used relatively low antibody thresholds, it is difficult to explore differences between BNT162b2 and mRNA-1273 induced immunity. As strong, antibody-mediated neutralizing activity increases with higher vaccine-induced anti-S1-antibody levels, comprehensively and longitudinally quantifying a potential difference in the humoral immunogenicity resulting from the approved mRNA vaccines in IRD patients and examining the effects of immunomodulatory treatments thereon may help to optimize COVID-19 vaccination strategies for this vulnerable patient population. Our aim was, therefore, to carry out a long-term, model-based comparative analysis of the magnitude and kinetics of the humoral immune response following two-dose vaccination with BNT162b2 and mRNA-1273 in patients with IRD on different immunomodulatory treatments.

## Methods

### Study set-up and participants

Between 1 March and 30 September 2021, adult patients from the Swiss cohort for patients with IRD (SCQM, Swiss Clinical Quality Management) who planned to receive an mRNA COVID-19 vaccine and were active users of the mySCQM patient application (21) were recruited into the study. The Geneva Ethics Committee approved the study protocol (BASEC-ID: 2020-01708), and all participants provided written informed consent. Participants’ demographics and clinical characteristics were extracted from physician- and patient-reported data from the SCQM cohort database. In addition, at predefined intervals, patients were asked to answer study-specific questionnaires *via* the patient app. These included questions regarding testing for active SARS-CoV-2 infections (if any), COVID-19 vaccination details, changes in medication intake, pausing of immunomodulatory therapies around the vaccination dates, and serious vaccine-related adverse events. The detailed study schedule and questionnaire are available in **Supplementary Figure S1** and **Table S1**, respectively. Participants received blood collection kits for the self-collection of capillary blood samples (Labonovum, NL), along with instructions for use. Participants were required to collect samples at baseline (i.e., before the first vaccine dose) and 4, 12, and 24 weeks post the second vaccine dose. Some patients with a past SARS-CoV-2 infection, were only given a single dose of an mRNA vaccine according to the Swiss immunization recommendations; others received two doses despite their previous infection. Samples were sent to the centralized laboratory in Geneva with a maximum allowed storage of 2 days at 2-8 °C before shipping and a postal time of ≤ 24 hours to ensure that anti-SARS-CoV-2 spike IgG antibodies were stable in the samples upon reception by the laboratory (22). Samples were tested for IgG antibodies against the S1 domain of the spike protein of SARS-CoV-2 using the EUROIMMUN ELISA. The assay read-out is a unitless index, calculated as the ratio of the optical density of the sample over that of the calibrator. We applied previously validated cut-offs: indices < 0.8 were considered negative, those ≥ 0.8 < 2.5 indeterminate and subsequently confirmed positive/negative with recombinant immunofluorescence (those ≥2.5 considered positive) (23). Prior SARS-CoV-2 infection was defined by records of a past positive anti-SARS-CoV-2 IgG or PCR test in the SCQM cohort database or a positive baseline anti-SARS-CoV2 IgG result.

### Final dataset for analysis

Only patients who received an mRNA COVID-19 vaccine, who provided an eligible baseline sample plus at least one subsequent sample, who fully answered the study questionnaires, and for whom the data regarding demographics and clinical characteristics extracted from the SCQM registry database were complete, were included in the analysis. Samples were considered eligible if enough serum for the assay could be extracted and if they were collected within the window of predefined collection timepoints (**Figure S2**). Samples taken after breakthrough infections or after receiving additional vaccine doses were excluded from the analysis. Samples not yet collected/tested at the time of writing were also not included in the analysis.

### Outcomes and objectives

The anti-SARS-CoV-2 spike IgG levels (anti-S1; expressed as a unitless optical density ratio) at 4, 12, and 24 weeks post second vaccine dose were our outcomes of interest. The primary and secondary study objectives were to compare these outcomes depending on the vaccine received (BNT162b2 vs mRNA-1273) and the immunomodulatory treatment at baseline while adjusting for age, disease, and past SARS-CoV-2 infection.

### Statistical methods

Models: We applied mixed-effects continuous outcome logistic regression models to the anti-S1 levels obtained at 4, 12, and 24 weeks post second vaccine dose to analyze differences depending on the vaccine and immunomodulatory treatment while accounting for inter-lot and inter-batch variability. These models are appropriate for the ELISA output, which is bounded (by a lower bound > 0 and an upper saturation limit), and, with the assumption of proportional odds, permit the comparison of the immunogenicity, following different vaccines and treatments, in relation to a given antibody cut-off, without the need to predefine it (24). Specifically, at each timepoint considered and for the covariates included, these models return the odds ratios of the vaccine-induced antibody levels being higher than a given cut-off without needing to pre-specify it. This is important since, to date, no absolute correlate of protection against severe COVID-19 has been established, and cut-offs may shift with the emergence of new variants (25). Since the true optical density ratios at the assay upper saturation limit are higher than this limit by an unknown amount, which we did not quantify through sample dilution, we treated the few observations at the saturation limit as right-censored.

Covariates: The following covariates assessed at baseline were included in the models applied at each timepoint: age, disease, past-SARS-CoV-2 infection, vaccine, immunomodulatory treatment as mono/combination therapy, as well as the interaction of vaccine with age (the odds ratios reported are therefore adjusted). Multiple other interactions were investigated prior to the final modelling, including those between vaccine and treatment, but only significant ones were retained in the final model. The majority (89%) of the study population indicated no reduction or pause in their immunomodulatory therapy during vaccination. Therefore, we decided not to include treatment changes in the analysis.

Confounding: In Switzerland, the BNT162b2 vaccine rollout began before that of mRNA-1273, at a time when the vaccination of the elderly and immunocompromized was prioritized. By including age and treatment as covariates in the model, we adjusted for this potential confounding of the vaccine effect by the timing of the vaccination.

Contextualization of absolute antibody levels: In addition to the relative comparisons emerging from the application of the models mentioned above, we sought to contextualize our results as it has been demonstrated that anti-S1 levels expressed as optical density ratios ≥ 5 (using the same EUROIMMUN assay) allowed to identify sera from SARS-CoV-2 convalescent plasma donors with strong neutralizing capacity (90% inhibition ﻿plaque reduction neutralization test (PRNT90) titers ≥ 1:20) with high specificity (26). Accordingly, at the different timepoints considered, we compared the proportion of SARS-CoV-2 naïve BNT162b2 and mRNA-1273 recipients with optical density ratios equal to or greater than this cut-off.

## Results

Between 4 March and 16 September 2021, 917 patients consented to participate in the study (**Table S2**). Five hundred and sixty-five patients received an mRNA COVID-19 vaccine, provided eligible samples and had complete data (**Figure S3** and **Table 1**). The total number of eligible samples that were tested were 565, 552, 542, and 513 at baseline, 4, 12, and 24 weeks post second vaccine dose, respectively (**Figure S3**). At the 4-, 12-, and 24-week post-second vaccine dose timepoints, only 3.6%, 1.5%, and 1.2% of the optical density ratios, respectively, were at the upper saturation limit and were treated as right-censored. BNT162b2 and mRNA-1273 recipients had comparable demographics and clinical characteristics (**Table 1**).

**Table 1:** Demographics and clinical characteristics of the study population.

	Total (n = 565)	BNT162b2 (n = 305)	mRNA-1273 (n = 260)
**Age at baseline, years [median (IQR)]**	53 (44 – 62)	54 (43 – 62)	52 (45 – 61)
**Sex, n (%)**			
** Female**	374 (66)	204 (67)	170 (65)
** Male**	191 (34)	101 (33)	90 (35)
**Disease duration at baseline, years [median (IQR)]**	15 (8 – 22)	14 (8 – 22)	15 (8 – 21)
**Evidence of SARS-CoV-2 infection, n (%)**			
** Past infection**	58 (10)	30 (10)	28 (11)
** No past infection**	507 (90)	275 (90)	232 (89)
**Vaccine, n (%)**			
** BNT162b2; mRNA-1273**	305 (54); 260 (46)	305 (100)	260 (100)
of which one dose*	*4; 7*	*4*	*7*
** interval between doses [median (IQR)]**	28 (28 – 29)	28 (28 – 29)	28 (28 – 29)
**Diagnosis, n (%)**			
** Rheumatoid arthritis**	204 (36.1)	112 (36.7)	92 (35.4)
** Axial spondyloarthritis**	207 (36.6)	107 (35.1)	100 (38.5)
** Psoriatic arthritis**	120 (21.2)	62 (20.3)	58 (22.3)
** Undifferentiated arthritis**	34 (6.0)	24 (7.9)	10 (3.8)
**Treatment at baseline, n (%)**			
** no medication**	84 (14.9)	42 (13.8)	42 (16.2)
** csDMARD**	52 (9.2)	23 (7.5)	29 (11.2)
*** of which combination therapy with GC***	*4*	*1*	*3*
** GC monotherapy**	5 (0.9)	4 (1.3)	1 (0.4)
** TNFi**	273 (48.3)	152 (49.8)	121 (46.5)
*** of which combination therapy* ^±^ *, n***	*76*	*47*	*29*
** JAKi**	36 (6.4)	21 (6.9)	15 (5.8)
*** of which combination therapy* ^⊥^ *, n***	*12*	*7*	*5*
** IL-6/17/23i**	77 (13.6)	45 (14.8)	32 (12.3)
*** of which combination therapy* ^⊥^ *, n***	*20*	*11*	*9*
** Rituximab**	20 (3.5)	9 (3.0)	11 (4.2)
*** of which combination therapy* ^⊥^ *, n***	*10*	*5*	*5*
** time since last infusion, days [median (IQR)]**	267 (179 – 568)	262 (215 – 372)	286 (142 – 706)
** Abatacept**	14 (2.5)	8 (2.6)	6 (2.3)
*** of which combination therapy* ^⊥^ *, n***	*6*	*5*	*1*
** PDE4i**	4 (0.7)	1 (0.3)	3 (1.2)
*** of which combination therapy* ^×^ *, n***	*1*	*-*	*1*
**Total GC use over all patients**			
**in mono- and combination therapy, n (%)** **Dose, mg [median (IQR)]**	32 (5.7) 5 (2.5 - 7.5)	20 (6.6) 5 (2.5 - 6)	12 (4.2) 7.5 (5 – 10)

*Due to past SARS-CoV-2 infection. No medication, currently on no immunomodulatory medication; csDMARD, conventional synthetic disease-modifying antirheumatic drug; GC, glucocorticoids; TNFi, tumor necrosis factor inhibitor; JAKi, Janus kinase inhibitor; IL-6/17/23i, interleukin 6/17/23 inhibitors; PDE4i, phosphodiesterase-4 inhibitor. ^±^with csDMARD/GC/csDMARD & GC; ^⟂^with csDMARD/csDMARD & GC; ^×^with csDMARD. The 32 patients receiving GCs are double counted, i.e. included in individual treatment groups and the entire GC group, to show the extent of GC use over the entire study population.

**Figure 1:**
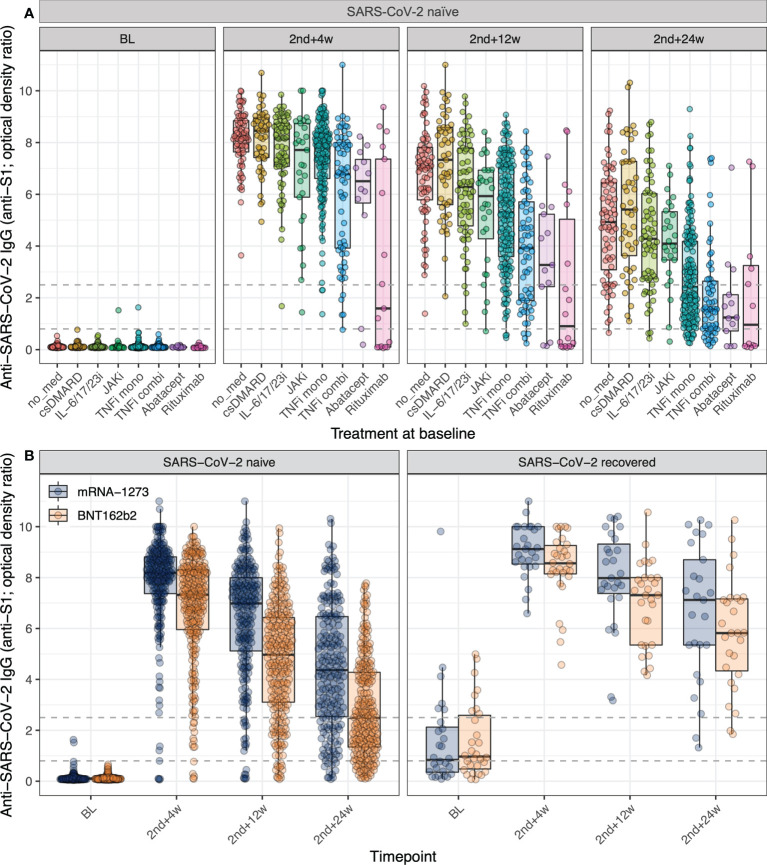
Impact of treatment for IRD and mRNA COVID-19 vaccine on anti-S1 antibody levels. **(A)** The variation over time of anti-S1 antibodies post mRNA COVID-19 vaccination in adult SARS-CoV-2 naïve IRD patients disaggregated by treatment group at baseline. No med = currently on no medication; csDMARD = conventional synthetic disease-modifying antirheumatic drugs in mono or combination therapy with GC (glucocorticoids); IL-6/17/23i = interleukin 6/17/23 inhibitors in mono or combination therapy with csDMARD/csDMARD & GC; JAKi = janus kinase inhibitors in mono or combination therapy with csDMARD/csDMARD & GC; TNFi mono = tumor necrosis factor inhibitor as monotherapy, TNFi combi = TNFi in combination therapy with csDMARD/GC/csDMARD & GC; Abatacept in mono or combination therapy with csDMARD/csDMARD & GC; Rituximab in mono or combination therapy with csDMARD/csDMARD & GC. The following treatment groups with five or fewer participants are not shown here: GC monotherapy and PDE4i (phosphodiesterase-4 inhibitor) in mono or combination therapy with csDMARD. **(B)** The variation over time of anti-S1 antibodies post mRNA COVID-19 vaccination in adult IRD patients disaggregated by vaccine and evidence of SARS-CoV-2 infection. For both panels: The dashed lines indicate the assay thresholds (see Methods). Individual points are overlaid on boxplots, with whiskers extending to 1.5*IQR. BL = baseline (day of 1^st^ vaccine dose, before vaccination), 2nd+4w/12w/24w = 4/12/24 weeks post 2^nd^ vaccine dose. For the full, adjusted model outcomes, see **Table 2**.

We analyzed how participants’ treatments affected humoral immunogenicity in SARS-CoV-2 naïve IRD patients (**Figure 1A**; **Table 2**). As monotherapy, the use of abatacept, JAKi, rituximab, and TNFi resulted in significantly lower antibody levels compared to those observed in the group of patients who were not on immunomodulatory medication at baseline (**Table 2**), with the latter group of patients currently not on medication presenting anti-S1 levels comparable to those previously reported for healthy individuals (**Figure S4**). Of note, compared to the untreated IRD group, monotherapy with biologics targeting other cytokines than TNF (i.e. IL-6/17/23i) did not negatively affect the humoral immune response (**Table 2**). In combination therapy, interleukin inhibitors and TNFi led to significantly lower antibody levels than respective monotherapy over all timepoints. **Table S3** provides the summary statistics (median, range and IQR) of the absolute antibody levels expressed as optical density ratios for each medication group and timepoint.

**Table 2:** The (adjusted) odds ratios of higher antibody levels (regardless of the threshold) up to 24 weeks post 2^nd^ vaccine dose for IRD patients.

Weeks post 2^nd^ vacc. dose: (Total number of samples available for analysis)	4 (552)		12 (542)		24 (513)	
	OR (95% CI)	p-value	OR (95% CI)	p-value	OR (95% CI)	p-value
**Age^×^**	**0.96** (0.94 - 0.97)	< 0.0001	**0.98** (0.96 - 0.99)	0.0045	0.99 (0.97 -1.0)	0.074
**mRNA-1273 vs BNT162b2**	**3.3** (2.4 - 4.6)	< 0.0001	**3.8** (2.7 - 5.4)	< 0.0001	**3.8** (2.7 - 5.2)	< 0.0001
**Past SARS-CoV-2 infection vs none**	**8.2** (4.8 - 14)	< 0.0001	**8.6** (5.1 - 15)	< 0.0001	**13** (7.2 - 22)	< 0.0001
Abatacept monotherapy*	**0.13** (0.035 - 0.45)	0.0013	**0.081** (0.020 - 0.32)	0.00036	**0.082** (0.021 - 0.32)	0.00034
cDMARD monotherapy*	1.3 (0.66 - 2.4)	0.49	1.8 (0.93 - 3.4)	0.083	**2.2** (1.1 - 4.4)	0.022
IL-6/17/23i monotherapy*	0.97 (0.54 - 1.7)	0.92	1.0 (0.55 - 1.9)	0.97	1.0 (0.56 - 1.9)	0.95
JAKi monotherapy*	**0.37** (0.16 - 0.84)	0.018	**0.36** (0.15 - 0.85)	0.020	0.64 (0.27 - 1.5)	0.29
Rituximab monotherapy*	**0.12** (0.022 - 0.62)	0.012	**0.074** (0.014 - 0.40)	0.0025	**0.11** (0.025 - 0.51)	0.0046
TNFi monotherapy*	**0.41** (0.26 - 0.65)	0.00014	**0.28** (0.18 - 0.45)	< 0.0001	**0.16** (0.098 - 0.26)	< 0.0001
**RA vs axSpA**	1.0 (0.65 - 1.5)	0.98	1.1 (0.72 - 1.7)	0.68	0.89 (0.58 - 1.5)	0.59
**PsA vs axSpA**	0.98 (0.64 - 1.5)	0.93	0.94 (0.61 - 1.4)	0.78	0.95 (0.61 - 1.5)	0.82
**UA vs axSpA**	0.90 (0.48 - 1.7)	0.75	1.3 (0.65 - 2.4)	0.50	1.0 (0.53 – 1.9)	0.98
**Abatacept combi^#^**	0.97 (0.14 - 6.8)	0.98	0.49 (0.070 - 3.5)	0.48	0.36 (0.055 - 2.4)	0.29
**IL-6/17/23i combi^#^**	**0.34** (0.12 - 0.95)	0.039	**0.26** (0.094 - 0.73)	0.011	**0.24** (0.086 - 0.68)	0.0070
**JAKi combi^#^**	1.2 (0.31 - 4.9)	0.77	1.2 (0.37 - 4.0)	0.75	1.1 (0.35 - 3.6)	0.85
**Rituximab combi^#^**	**0.044** (0.0051 - 0.37)	0.0040	**0.091** (0.012 - 0.71)	0.022	0.14**^§^** (0.015- 1.3)	0.087**^§^**
**TNFi combi^±#^**	**0.39** (0.23 - 0.65)	0.00039	**0.36** (0.22 - 0.60)	< 0.0001	**0.38** (0.22 - 0.64)	0.00029
**Interaction of age with vaccine^+^**	**1.05** (1.02 - 1.07)	0.00078	1.02 (0.995 - 1.05)	0.11	**1.03** (1.01 - 1.06)	0.018

^×^For 1 y increase with BNT162b2. The following treatments are considered at baseline: *vs no medication (= currently on no medication). csDMARD, conventional synthetic disease-modifying antirheumatic drugs; IL-6/17/23i, interleukin 6/17/23 inhibitors; JAKi, janus kinase inhibitors; TNFi, tumor necrosis factor inhibitors. RA, rheumatoid arthritis; axSpA, axial spondyloarthritis; PsA, psoriatic arthritis; UA, undifferentiated arthritis. Combi, combination therapy with csDMARD/csDMARD & GC; ±combination therapy with csDMARD/GC/csDMARD & GC; #vs respective monotherapy ^+^Interaction term showing how the OR of mRNA-1273 vs BNT162b2 increased with increasing age (indicatively, at 4 and 24 weeks post 2nd vaccine dose, for every increase in age of 1 year, the odds ratio of higher antibody levels with mRNA-1273 vs BNT162b2 increased by 5% and 3%, respectively). The following treatment groups with five or fewer participants were included in the model but are not shown here: GC (glucocorticoid) monotherapy, PDE4i (phosphodiesterase-4 inhibitor) monotherapy and combination therapy, and csDMARD combination therapy. ^§^At the time of analysis, only five samples from participants in this group were available from this timepoint.

Bold values indicate odds ratio estimates that were statistically significant.

**Figure 2:**
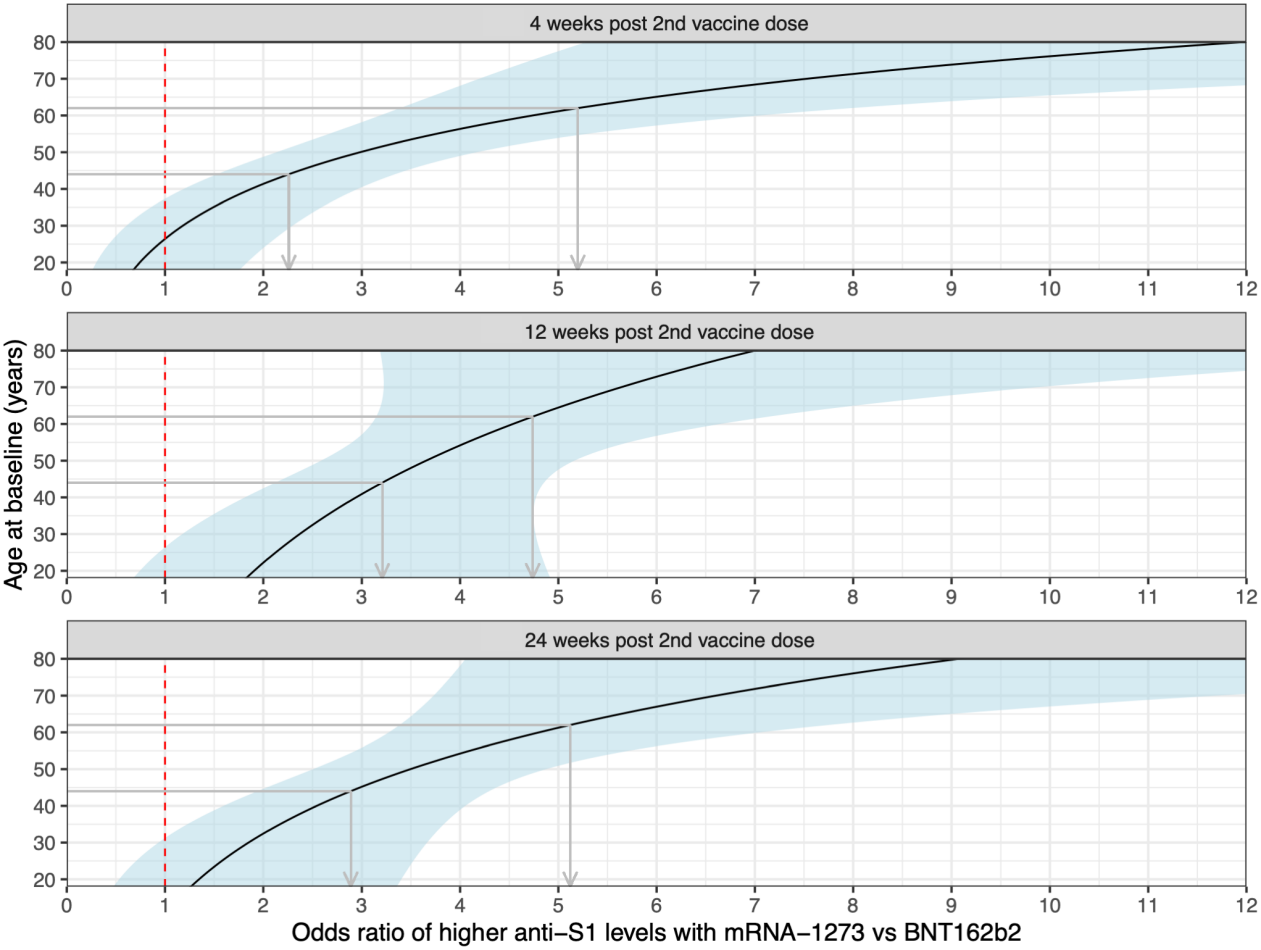
Age-dependent odds of higher antibody levels following mRNA-1273 vs BNT162b2 vaccination at the different timepoints. The dashed red line indicates the location of odds ratio = 1, and the light blue-shaded areas the pointwise 95% confidence intervals. The grey arrows demonstrate, at the different timepoints, the increased benefit (in terms of higher odds of higher antibody levels) from vaccination with mRNA-1273 vs BNT162b2 for an IRD patient of 62 y vs 44 y (an interval coinciding with the study population age IQR).

Comparing the humoral immunogenicity of the two administered mRNA vaccines (**Figure 1B**; **Table 2**), we observed that the odds of having higher antibody levels than any given threshold at 4, 12, and 24 weeks post second vaccine dose were, respectively, 3.4, 3.8, and 3.8 times higher following vaccination with mRNA-1273 compared to BNT162b2 for the average-aged patient (53 y) in this study (p < 0.0001, **Table 2**). This was irrespective of the disease, immunomodulatory treatment, and past SARS-CoV-2 infection. Moreover, for every one-year increase in age, the odds ratio of higher peak antibody levels following mRNA-1273 versus BNT162b2 vaccination increased by 5% (age – vaccine interaction at 4 weeks post second vaccine dose, p < 0.001, **Table 2**). This effect was cumulative (**Figure 2**, panel ‘4 weeks post 2nd vaccine dose’) ratio of higher peak antibody levels with mRNA-1273 versus BNT162b2 were over two times greater for patients ≥ 62 y (the eldest 25% of the population) versus patients ≤ 44 y (the youngest 25% of the population; **Figure 2**, panel ‘4 weeks post 2nd vaccine dose’). Vaccination of patients with a past SARS-CoV-2 infection led to strikingly higher peak antibody levels than in SARS-CoV-2 naïve IRD patients (odd ratios 8.2, 8.6, and 13 at 4, 12, and 24 weeks post second vaccine dose, respectively; p < 0.0001, **Table 2**). **Table S4** provides the summary statistics (median, range and IQR) of the absolute antibody levels in optical density ratios for each vaccine, SARS-CoV-2 infection status, and timepoint.

In spite of the independence of the model-based results from any priorly defined cut-off, the absolute antibody levels (**Figure 1**; **Tables S3**, **S4**) can also be contextualized in terms of the optical density ratio cut-off of 5, which has previously been described to identify sera from SARS-CoV-2 convalescent plasma donors with strong neutralizing capacity against the original Wuhan strain of SARS-CoV2 (26). We observed differences in the proportion of SARS-CoV-2 naïve patients with sera reaching an optical density ratio of ≥ 5 in recipients of mRNA-1273 compared to BNT162b2. Specifically, the proportion of samples with an optical density ratio ≥ 5 from mRNA-1273 recipients was 93%, 75%, and 40% at 4, 12, and 24 weeks post second vaccine dose, whereas, during the same respective period, this was only the case in 84%, 50%, and 15% of samples from BNT162b2 recipients (**Figure 1B**, SARS-CoV-2 naïve patients). While potentially not applicable to the neutralization of more recent variants of concern, this finding suggests that it can be assumed that the higher absolute antibody levels were associated with more robust virus neutralization.

## Discussion

Longitudinal data on anti-SARS-CoV-2 vaccine-induced immune responses beyond three months for patients on immunomodulatory therapies are still limited but of high clinical relevance given the widespread use of the latter in a variety of immune-mediated diseases. Our results confirmed differences in vaccine-induced antibody responses driven by distinct treatment modalities as others have reported (4, 5, 7, 27, 28). Impaired antibody responses were observed in patients on rituximab, abatacept, TNFi and JAKi supporting the view that the formation of a robust vaccine-induced immune response depends on complex interactions between distinct components of the immune system that may be differentially affected depending on the mode of action of the treatment used. Of note, irrespective of the underlying diagnosis and of the treatment modality, we observed higher odds of increased anti-S1 antibody levels at all timepoints following vaccination with mRNA-1273 versus BNT162b2 in the patients of our cohort. Moreover, our results suggest that in IRD patients the benefit – in terms of humoral immune response – of vaccination with mRNA-1273 versus BNT162b2 increases with age. The higher humoral immunogenicity could be due to the higher dose of mRNA in the mRNA-1273 vaccine, differences in the immune activation by the proprietary mRNA modifications introduced by each manufacturer, or a combination thereof (29). In Switzerland, both vaccines were given at a four weeks interval, excluding a difference introduced by dosing schedules.

The observation that the effect of higher antibodies in mRNA-1273- vs BNT162b2-vaccinated patients increased with age is of special interest. Age is an important factor for reduced immunogenicity and vaccine-induced protection due to immunosenescence (30). Higher antigen doses of influenza or hepatitis B vaccines enable to increase vaccine responses in older adults or patients at risk for vaccine non response (31, 32). Therefore, it can be speculated that the higher dose of the mRNA-1273 may overcome an age-related decrease in immunogenicity. Indeed, for an increase in age of 10 years, the odds of higher antibody levels following BNT162b2 vaccination at peak immunogenicity decreased by a factor of 0.65 (95% CI 0.54, 0.77), while following mRNA-1273 vaccination the change in the odds with 10 more years was estimated as 1.02 (95% CI: 0.83, 1.25). With BNT162b2 therefore, we have evidence that the odds decrease with age (confidence interval entirely below 1), whereas with mRNA-1273 the odds decrease at a lower rate with age compared to BNT162b2, or might even increase (confidence interval includes 1 and does not overlap with the confidence interval for BNT162b2). Consequently, we observe an increasing benefit of mRNA-1273 over BNT162b2 with age.

Strong vaccine-induced anti-S1 antibody responses have been shown to correspond to neutralization of viral variants and correlated with a better clinical outcome (33–35). Yet, to date, no anti-S1 cut-off has been established that correlates with protection from severe COVID-19. Moreover, higher antibody levels are deemed necessary to protect against different variants of concern compared to the wild-type virus (36). With the assumption of proportional odds, the statistical models applied in this study permit the effective comparison of the impact of different mRNA vaccines and treatments on the antibody levels within a bounded range, without the need to predefine a formal cut-off (24). Furthermore, the applied models also enable adjusting for confounding, allowing for an informative comparison. To our knowledge, a longitudinal comparison of the humoral immunogenicity following two-dose mRNA COVID-19 vaccination in patients with rheumatic diseases has not been performed in this level of detail to date, with relevant studies reporting results in terms of the proportion of individuals achieving seroconversion or passing a relatively low antibody threshold, making it difficult to investigate differences between the BNT162b2 and mRNA-1273 vaccines (5, 19, 20).

Despite the independence of the model-based results from any priorly defined cut-off, the absolute optical density ratios were also contextualized in terms of the cut-off of 5, that has been demonstrated to correspond to strong neutralization capacity against the original Wuhan strain of SARS-CoV2 in plasma derived from convalescent patients (26). While this cut-off is potentially not applicable to the neutralization of more recent variants of concern, the comparison of the proportion of samples per vaccine passing this threshold suggests a higher neutralization capacity of mRNA-1273 compared to BNT162b2. Specifically, while the proportion of SARS-CoV-2 naïve mRNA-1273 and BNT162b2 recipients with antibody levels equal to or above this cut-off is comparable at the timepoint of peak immunogenicity (93% versus 84%, respectively), by 12 and 24 weeks post second vaccine dose 25% more mRNA-1273 recipients achieve this antibody cut-off compared to BNT162b2 recipients. Unlike the adjusted statistical models that form the principal analysis in this study, the comparison in terms of proportions of patients achieving this cut-off is unadjusted. Nevertheless, it is worth noting that the demographic and clinical characteristics of both vaccine recipients were comparable (**Table 1**).

The limitations of this study include the sampling by capillary blood self-collection, which, while maximizing participation due to its convenience, provided limited serum volumes and restricted the assay choice to the semi-quantitative EUROIMMUN ELISA that requires a small amount of serum. Nevertheless, in terms of accuracy of results for a series of biomarkers, capillary blood self-sampling did not suffer compared to venous blood draws, as demonstrated by a recent randomized controlled trial (37). Another limitation of this study was the inability to perform neutralization or cellular assays or measure antibody responses to other SARS-CoV-2 antigens. Consequently, we have no data on how the vaccination-induced T cell responses compare between the two mRNA vaccines in patients with IRD. Interestingly, in a recent study on RA patients vaccinated with BNT162b2, significant impairment of the humoral immune response but less so of the T cell response was found at six months post vaccination (38). Moreover, we did not include a matched healthy control group, although we were able to establish that the group of IRD patients on no immunomodulatory medication at baseline had vaccine-induced antibody responses comparable to those reported for healthy individuals (**Figure S4**). Finally, the study results warrant a comparison of potential vaccine-associated side effects, which were not systematically captured in this study (the questionnaire involved questions on severe vaccine-related adverse events only; **Table S1**). We were also unable to systematically assess disease activity, however recent studies support the safety of mRNA anti-SARS-CoV2 vaccines in patients and report on infrequent flares of the underlying rheumatic disease (39, 40).

In conclusion, our results suggest that in patients with IRD, who are at risk of a poor vaccine response, two-dose vaccination with mRNA-1273 versus BNT162b2 results in higher peak anti-S1 levels, even more so in elderly patients, and longer antibody persistence. Immunogenicity is only a potential surrogate for vaccine effectiveness and future studies will show whether the observed difference in immunogenicity has an impact on breakthrough infections and whether it persists or levels out after further mRNA COVID-19 vaccine doses.

## Data availability statement

Study-related data can be made available from the SCQM Foundation according to the SCQM Rules of Research after the publication of all study-related research objectives. Researchers interested in further analyzing the data resulting from this study can contact the SCQM Foundation (scqm@hin.ch). Data can only be used for scientific research. SCQM is an ongoing, long-term registry with no end date for data collection and data provision.

## Ethics statement

This study involving human participants was reviewed and approved by the Geneva Ethics Committee. Patients provided their written informed consent to participate in this study.

## Author contributions

CR was involved in project administration, funding acquisition, supervision, conceptualization, investigation, methodology, formal analysis, visualization, and writing the original manuscript draft. CB was involved in the study conceptualization, formal analysis, and writing of the original manuscript draft. AC was involved in funding acquisition, conceptualization, and reviewing & editing of the manuscript. CP was involved in data curation, software, methodology, formal analysis, visualization, and reviewing & editing of the manuscript. DA, PL, and NV contributed to resources, investigation, validation, and reviewing & editing of the manuscript. TM contributed to project administration, supervision, funding acquisition, resources, investigation, data curation, and reviewing & editing of the manuscript. MR was involved in conceptualization, formal analysis, methodology, and reviewing & editing of the manuscript. AS contributed to the conceptualization, and reviewing & editing of the manuscript. IvL and JS were patient representatives involved in conceptualization and reviewing & editing of the manuscript. KL, BM, and AF contributed to conceptualization, and reviewing & editing of the manuscript. AR-R was the principal investigator involved in funding acquisition, study conceptualization, formal analysis, and writing the original manuscript draft. All authors contributed to the article and approved the submitted version.

## Funding

This study was investigator-initiated and received independent financial support from an anonymous donation from a research foundation and Moderna Switzerland GmbH. The study sponsors had no role in the study design or in the collection, analysis and interpretation of the data, the writing of the manuscript or the decision to submit the manuscript for publication. The following pharmaceutical industries financially support the SCQM: Abbvie, iQone Healthcare, Janssen, Eli Lilly, MSD Merck Sharp & Dohme, Novartis, Pfizer, Samsung Bioepis, Sandoz, and Biogen.

## Acknowledgments

A list of rheumatology offices and hospitals contributing to the SCQM registries can be found on www.scqm.ch/institutions. The SCQM thanks the patients for their participation in this study.

## Conflict of interest

KL reports personal fees from Gilead-Galapagos, Pfizer, Viatris, Celltrion, outside of the submitted work. AF has received research support from Abbvie, Eli-Lilly, Galapagos, and Pfizer outside of the submitted work, and consultancies or speaker honoraria for Abbvie, BMS, Eli-Lilly, Gilead, Pfizer, Sanofi, and UCB outside of the submitted work. AR-R reports honoraria for consultation and lectures from Abbvie, Amgen, Pfizer, Gilead, Novartis, Janssen, Eli-Lilly, Sanofi, Roche, UCB, BMS outside of the submitted work.

The remaining authors declare that the research was conducted in the absence of any commercial or financial relationships that could be construed as a potential conflict of interest.

## Publisher’s note

All claims expressed in this article are solely those of the authors and do not necessarily represent those of their affiliated organizations, or those of the publisher, the editors and the reviewers. Any product that may be evaluated in this article, or claim that may be made by its manufacturer, is not guaranteed or endorsed by the publisher.
