# Supplementary material for: Type of mRNA COVID-19 vaccine and immunomodulatory treatment influence humoral immunogenicity in patients with inflammatory rheumatic diseases
Source: Front Immunol. 2022 Oct 13;13:1016927. doi: 10.3389/fimmu.2022.1016927 (PMC9606233; doi:10.3389/fimmu.2022.1016927)
Supplement: Supplementary file 1 [file DataSheet_1.docx]

**Supplementary Material**

Increased anti-SARS-CoV2 humoral immunogenicity following vaccination with mRNA-1273 versus BNT162b2 in patients with inflammatory rheumatic diseases

Catherine Elizabeth Raptis^1^, Christoph Tobias Berger^2,3^, Adrian Ciurea^4^, Diego Olivier Andrey^5,6^, Christos Polysopoulos^1^, Pierre Lescuyer^5^, Tanja Maletic^1^, Myriam Riek^1^, Almut Scherer^1^, Isabell von Loga^1^, Judith Safford^7^, Kim Lauper^8,6^, Burkhard Möller^9^, Nicolas Vuilleumier^5,6^, Axel Finckh^8,6^, Andrea Rubbert-Roth*^10^

^1^SCQM Foundation, Zurich, Switzerland, ^2^University Center for Immunology and Immunization Clinic, University Hospital Basel, Basel, Switzerland, ^3^Translational Immunology, Department of Biomedicine, University of Basel, Basel, Switzerland, ^4^Department of Rheumatology, Zurich University Hospital, University of Zurich, Zurich, Switzerland, ^5^Laboratory Medicine Division, Geneva University Hospitals, Geneva, Switzerland, ^6^Faculty of Medicine, University of Geneva, Geneva, Switzerland, ^7^RheumaCura, Bern, Switzerland, ^8^Division of Rheumatology, Geneva University Hospitals, ^9^Division of Rheumatology and Immunology, Inselspital, Bern University Hospital, Bern, Switzerland, ^10^Division of Rheumatology and Immunology, St. Gallen Cantonal Hospital, St. Gallen, Switzerland

*Corresponding author email address: Andrea.Rubbert-Roth@kssg.ch

# List of elements

| **Figure S1** | Participant study schedule |
| --- | --- |
| **Table S1** | Study questionnaire |
| **Figure S2** | Distribution of sample collection times per study timepoint |
| **Figure S3** | Study population tree |
| **Table S2** | The demographics and clinical characteristics of patients who signed up for the study |
| **Methods** S**1** | Comparison of antibody levels from IRD patients on no medication with that of healthy individuals |
| **Figure S4** | Application of simple exponential decay model to the antibody levels obtained from SARS-CoV-2 naïve IRD patients on no medication |
| **Table S3** | Summary statistics for antibody levels by treatment group |
| **Table S4** | Summary statistics for antibody levels by vaccine and SARS-CoV-2 infection status |

**Figure S1. The study schedule for participants of the study**, including timing of the self-blood collection and answering the study questionnaire via the patient app.

**Table S1. Study questionnaire.** The following questions appeared in the mySCQM app for patients participating in the study. Only patient with questionnaires that were fully answered by the time of writing were included in the analysis (i.e. there was no missingness in the final dataset). Additional data required for the analysis (e.g. demographics and clinical characteristics, including treatment at baseline) were extracted from the SCQM cohort database.

| # | Question |
| --- | --- |
| 1 | *For new mySCQM app users:*  Have you tested positive for SARS-CoV-2 since March 1, 2020?  (valid tests: PCR, antigen tests)  (invalid tests: antibody tests, self-tests).   - Yes *(🡪 continue to 2, 3)* / No / Unknown   *For existing mySCQM app users:*  Have you tested positive for SARS-CoV-2 since dd.mm.yyyy *(Date of last mySCQM entry)?*  (valid tests: PCR, antigen tests)  (invalid tests: antibody tests, self-tests).   - Yes *(🡪 continue to 2, 3)* / No / Unknown |
| 2 | When were you tested? (Please report on the most recent positive test)  What type of test was performed?   - Nasopharyngeal swab (PCR) / Nasopharyngeal swab (antigen) / Unknown |
| 3 | Were you hospitalized due to COVID-19?   - Yes / No / Unknown |
| 4 | Have you been vaccinated against COVID-19?   - Yes / No / Unknown / I do not wish to be vaccinated against COVID-19 - *In case no vaccination desired:* Please indicate why you do not wish to be vaccinated against COVID-19 (*free text*) - *If No/unknown*: *the patient sees question again until a different option is selected* - *If Yes:*   With which vaccine were you vaccinated against COVID-19? (You can find this information in your vaccination certificate)  Comirnaty (Pfizer/BioNTech) / COVID-19 Vaccine Moderna (Spikevax) / Other / unknown   - *If other:* Please enter the name of the vaccine: (*free text*) - Please enter the date of the 1st vaccination |
| 5 | Steroids before 1st vaccination against COVID-19.  Did you take cortisone medications such as prednisone, spiricort, or lodotra before your 1st vaccination against COVID-19?   - No - No, before the 1st vaccination I paused the cortisone medication   - For how long before the 1st vaccination did you pause the cortisone medication?   1 - 5 days before the 1st vaccination  6 - 9 days before the 1st vaccination  10 or more days before the 1st vaccination   - Yes, unchanged   ___ mg per day   - Yes, but before the 1st vaccination I reduced the cortisone medication   - How many mg of cortisone did you take per day?   ___ mg   - - - - For how long did you reduce the dose?   1 - 5 days before the 1st vaccination  6 - 9 days before the 1st vaccination  10 or more days before the 1st vaccination |
| 6 | Base therapy before 1st vaccination against COVID-19.  Did you adjust your base therapy prior to the 1st vaccination against COVID-19 because of the planned vaccination? The following DMARDs are considered as base therapy (*patient sees list of conventional synthetic/biologic/targeted synthetic DMARDs*)   - Yes (paused or reduced) / No / I do not take any DMARDs |
| 7 | Did you have any serious vaccine-related adverse events after your 1st vaccination against COVID-19 that were believed to be related to the vaccination and for which you required care by a healthcare professional?   - Yes / No / unknown   *If Yes:*  Describe the side effect in brief (f*ree text)* |
| 8 | Steroids after 1st vaccination against COVID-19.  Did you take cortisone medications such as prednisone, spiricort, or lodotra after your 1st vaccination against COVID-19?   - No - No, after the 1st vaccination I paused the cortisone medication   - For how long did you pause the cortisone medication?   1 - 5 days after the 1st vaccination  6 - 9 days after the 1st vaccination  10 or more days after the 1st vaccination   - Yes, unchanged   ___ mg per day   - Yes, but after the 1st vaccination I reduced the cortisone medication   - How many mg of cortisone did you take per day?   ___ mg   - - - - For how long did you reduce the dose?   1 - 5 days after the 1st vaccination  6 - 9 days after the 1st vaccination  10 or more days after the 1st vaccination |
| 9 | Base therapy after 1st vaccination against COVID-19.  Did you adjust your base therapy after the 1st vaccination against COVID-19 because of the vaccination? The following DMARDs are considered as base therapy (*patient sees list of conventional synthetic/biologic/targeted synthetic DMARDs*)   - Yes (paused or reduced) / No / I do not take any DMARDs |
| 10 | Have you received the 2nd vaccination against COVID-19?   - Yes / No / Unknown / Vaccination consists of only one dose   *If No/unknown: the patient sees question again until a different option is selected*  If Yes:  Please enter the date of the 2nd vaccination. |
| 11 | Steroids before 2^nd^ vaccination against COVID-19.  Did you take cortisone medications such as prednisone, spiricort, or lodotra before your 2^nd^ vaccination against COVID-19?   - No - No, before the 2^nd^ vaccination I paused the cortisone medication   - For how long before the 2^nd^ vaccination did you pause the cortisone medication?   1 - 5 days before the 2^nd^ vaccination  6 - 9 days before the 2^nd^ vaccination  10 or more days before the 2^nd^ vaccination   - Yes, unchanged   ___ mg per day   - Yes, but before the 2^nd^ vaccination I reduced the cortisone medication   - How many mg of cortisone did you take per day?   ___ mg   - - - - For how long did you reduce the dose?   1 - 5 days before the 2^nd^ vaccination  6 - 9 days before the 2^nd^ vaccination  10 or more days before the 2^nd^ vaccination |
| 12 | Base therapy before 2^nd^ vaccination against COVID-19.  Did you adjust your base therapy prior to the 2^nd^ vaccination against COVID-19 because of the vaccination? The following DMARDs are considered as base therapy (*patient sees list of conventional synthetic/biologic/targeted synthetic DMARDs*)   - Yes (paused or reduced) / No / I do not take any DMARDs |
| 13 | Did you have any serious vaccine-related adverse events after your 2^nd^ vaccination against COVID-19 that were believed to be related to the vaccination and for which you required care by a healthcare professional?   - Yes / No / unknown   *If Yes*:  Describe the side effect in brief (f*ree text*) |
| 14 | Steroids after 2^nd^ vaccination against COVID-19.  Did you take cortisone medications such as prednisone, spiricort, or lodotra after your 2^nd^ vaccination against COVID-19?   - No - No, after the 1st vaccination I paused the cortisone medication   - For how long did you pause the cortisone medication?   1 - 5 days after the 2^nd^ vaccination  6 - 9 days after the 2^nd^ vaccination  10 or more days after the 2^nd^ vaccination   - Yes, unchanged   ___ mg per day   - Yes, but after the 2^nd^ vaccination I reduced the cortisone medication   - How many mg of cortisone did you take per day?   ___ mg   - - - - For how long did you reduce the dose?   1 - 5 days after the 2^nd^ vaccination  6 - 9 days after the 2^nd^ vaccination  10 or more days after the 2^nd^ vaccination |
| 15 | Base therapy after 2^nd^ vaccination against COVID-19.  Did you adjust your base therapy after the 2^nd^ vaccination against COVID-19 because of the vaccination? The following DMARDs are considered as base therapy (*patient sees list of conventional synthetic/biologic/targeted synthetic DMARDs*)   - Yes (paused or reduced) / No / I do not take any DMARDs |
| 16 | Have you received a 3^rd^ vaccination against COVID-19?   - Yes / No / I did not want to receive a 3^rd^ vaccination - *If No: the patient sees question again until a different option is selected* - If Yes:   With which vaccine were you vaccinated? (You can find this information in your vaccination certificate)  Comirnaty (Pfizer/BioNTech) / COVID-19 Vaccine Moderna (Spikevax) / Other / unknown   - *If Other:* Please enter the name of the vaccine: (*freetext*)   Please enter the date of the 3^rd^ vaccination |


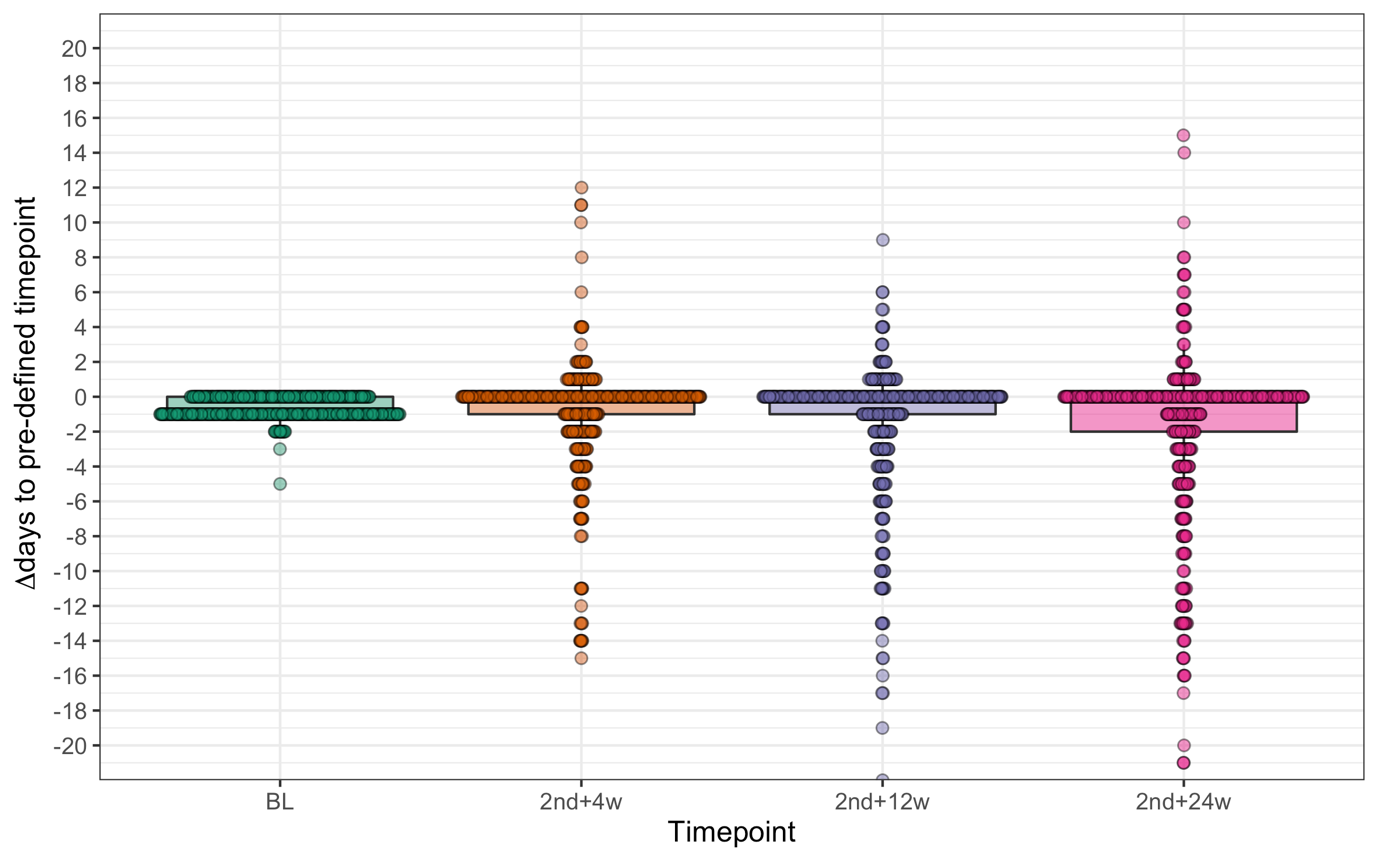


**Figure S2**. **Distribution of sample collection times per study timepoint.** Boxplots (with whiskers extending to 1.5*IQR) are overlaid by individual points. Results from samples with sufficient serum were considered eligible for inclusion in the analysis if they were collected within the following windows of the predefined collection timepoints: baseline samples (BL): taken up to seven days before first vaccine dose; samples at 4 weeks post second vaccine dose (2^nd^ + 4w): ± 14 days; samples at 12 weeks post second vaccine dose (2^nd^ + 12w): ± 21 days; samples at 24 weeks post second vaccine dose (2^nd^ + 24w): ± 28 days. A total of 565, 552, 542, and 513 eligible samples were analyzed from the collection timepoints at 4, 12, and 24 weeks post second vaccine dose, respectively. The shares of samples collected within ± 5 days of the predefined timepoints were: 100 % for baseline samples (up to 5 days *before* first vaccine dose); 93%, 90%, and 86% for samples collected 4, 12, and 24 weeks post second vaccine dose, respectively.

**Figure S3.** **Study population tree**. To be included in the analysis (final dataset), patients must have received an mRNA-based COVID-19 vaccine, they must have sent in an eligible baseline sample plus at least one subsequent eligible sample, and the data on their demographics and clinical characteristics must have been complete (no missingness). *All 592 patients fully answered the study questionnaire (no missingness), but for 28 of these patients the treatment at baseline could not be confirmed, hence their exclusion from the final dataset. Samples were considered eligible if enough serum for the assay could be extracted and if they were collected within the window of predefined collection time-points (Figure S2). Samples taken after breakthrough infections or further vaccine doses were excluded from the analysis. Samples not yet collected/tested at the time of writing were also not included in the analysis. **^+^**includes ‘no baseline sample sent’ or ‘only baseline sample sent’ or ‘only samples taken outside of pre-defined collection window’. The numbers in italics indicate the number of eligible samples tested and included in the analysis from each timepoint.

**Table S2**. **The demographics and clinical characteristics of patients who signed up for the study** (not all of whom were included in the analysis; see Figure S3).

| Population of patients who signed up for study (n = 917, 100%) | RA  (n = 303, 33%) | axSpA  (n = 364, 40%) | PsA  (n = 187, 20%) | UA  (n = 63, 7%) |
| --- | --- | --- | --- | --- |
| Age at baseline,  years (median (IQR)) | 57 (47 - 64) | 49 (39 - 57) | 55 (48 - 61) | 49 (38 - 59) |
| Sex, n (%) |  |  |  |  |
| female | 237 (78) | 195 (54) | 98 (52) | 44 (70) |
| male | 66 (21) | 169 (46) | 89 (48) | 19 (30) |
| Disease duration at baseline, years (median (IQR)) | 13 (8 - 20) | 17 (10 - 25) | 13 (8 - 20) | 10 (4 - 21) |
| Vaccine, n (%) |  |  |  |  |
| BNT162b2 | 163 (53.8) | 158 (43.4) | 87 (46.5) | 36 (57.1) |
| mRNA-1273 | 128 (42.2) | 169 (46.4) | 84 (44.9) | 21 (33.3) |
| other |  | 2 (0.5) |  |  |
| unknown |  | 3 (0.8) | 1 (0.5) | 1 (1.6) |
| NA (no vaccination recorded) | 12 (4) | 32 (8.8) | 15 (8) | 5 (7.9) |
| RA = rheumatoid arthritis; axSpA = axial spondyloarthritis; PsA = psoriatic arthritis; UA = undifferentiated arthritis. | | | | |

**Methods S1: Comparison of antibody levels from IRD patients on no medication with that of healthy individuals**

The group of IRD patients in the no medication group (currently not on medication: no DMARD and no GC, **n = 84, 65% female, median age 53 y (25^th^ percentile: 43 y, 75^th^ percentile: 61 y)**) served as a control group in our study. These patients mounted an antibody response that was comparable to that of healthy individuals of a similar sex ratio and age range, as demonstrated by comparison with data provided from a study by Moor and colleagues.(1) Using the same assay as used in this study, the authors measured the anti-S1 levels following mRNA COVID-19 vaccination in patients receiving CD20-targeted B cell-depleting treatment and in healthy controls (**n = 29, 66% female, median age = 54 y (25^th^ percentile: 45 y, 75^th^ percentile: 62 y)**). Samples from SARS-CoV-2 naïve healthy volunteers were collected at a median timepoint of 54 days post 2^nd^ vaccine dose. The median optical density ratio measured for these healthy controls was **7.34** (25th percentile: 6.44; 75th percentile: 8.00). To compare the antibody titres from this study’s group of SARS-CoV-2 naïve IRD patients on no treatment at the same timepoints, a simple exponential decay equation was applied to the data (Figure S4), of the form $Ab\left( t \right)={Ab}_{peak}e^{-\lambda t}$, where *Ab* = antibody level, *Ab_peak_* = peak antibody level (4 weeks post 2^nd^ vaccine dose), and *λ* = decay constant. Accordingly, the optical density ratio observed at 54 days post 2^nd^ vaccine dose was modelled to be **7.47**, a value comparable to **7.34** reported by Moor and colleagues, supporting the use of the group of IRD patients on no medication as a control with a humoral immune response similar to that of healthy controls.


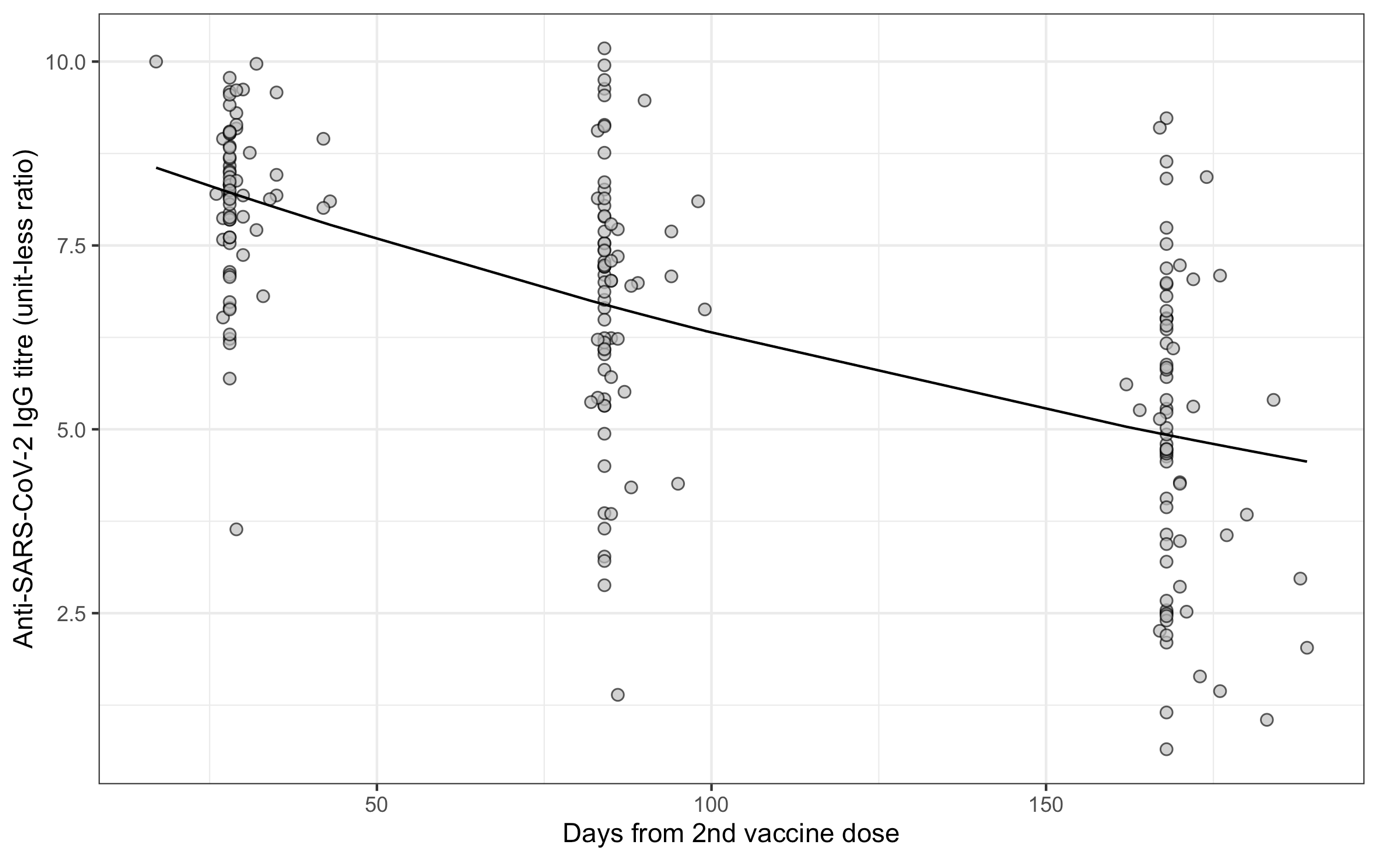


**Figure S4**. **Application of simple exponential decay model to the antibody levels obtained from SARS-CoV-2 naïve IRD patients on no medication** (no DMARD & no GC). Number of samples analyzed per timepoint: 82, 80, and 78 at 4, 12, and 24 weeks post second vaccine dose, respectively. Equation fitted via nonlinear least squares regression: $Ab\left( t \right)={Ab}_{peak}e^{-\lambda t}$, where *Ab* = antibody level, *Ab_peak_* = peak antibody level (4 weeks post 2^nd^ vaccine dose), *λ* = decay constant. By applying the fitted exponential decay equation, the optical density ratio observed at 54 days post 2^nd^ vaccine dose was modelled to be **7.47**, a value comparable to **7.34**, the median optical density ratio reported for the healthy control group at the same timepoint.^1^ See Methods S1, above, for further information.

**Table S3. Summary statistics for antibody levels by treatment group** for SARS-CoV-2 naïve IRD patients (antibody levels reported in terms of optical density ratios).

| **Timepoint** | **Treatment** | **Number of samples** | **Min.** | **1^st^ quartile** | **Median** | **3rd quartile** | **Max.** |
| --- | --- | --- | --- | --- | --- | --- | --- |
| BL | no_med | 76 | 0.060 | 0.090 | 0.11 | 0.14 | 0.53 |
|  | csDMARD | 50 | 0.060 | 0.080 | 0.10 | 0.14 | 0.77 |
|  | IL-6/17/23i | 69 | 0.060 | 0.080 | 0.10 | 0.13 | 0.55 |
|  | JAKi | 29 | 0.050 | 0.070 | 0.10 | 0.13 | 1.5 |
|  | TNFi mono | 176 | 0.050 | 0.080 | 0.10 | 0.13 | 1.6 |
|  | TNFi combi | 68 | 0.060 | 0.080 | 0.10 | 0.15 | 0.59 |
|  | Abatacept | 13 | 0.060 | 0.080 | 0.090 | 0.10 | 0.18 |
|  | Rituximab | 18 | 0.060 | 0.060 | 0.080 | 0.13 | 0.27 |
| 2nd+4w | no_med | 74 | 3.6 | 7.6 | 8.2 | 8.9 | 10 |
|  | csDMARD | 49 | 4.9 | 7.4 | 8.5 | 9.0 | 10.7 |
|  | IL-6/17/23i | 68 | 1.7 | 7.1 | 8.1 | 8.8 | 9.9 |
|  | JAKi | 29 | 1.4 | 5.9 | 7.7 | 8.7 | 10 |
|  | TNFi mono | 172 | 1.4 | 6.6 | 7.8 | 8.4 | 10 |
|  | TNFi combi | 66 | 0.77 | 3.9 | 6.8 | 8.0 | 11 |
|  | Abatacept | 12 | 0.19 | 5.5 | 6.5 | 7.4 | 8.2 |
|  | Rituximab | 17 | 0.060 | 0.10 | 1.6 | 7.4 | 9.4 |
| 2nd+12w | no_med | 72 | 1.4 | 5.8 | 7.0 | 7.8 | 10.2 |
|  | csDMARD | 49 | 2.1 | 5.6 | 7.3 | 8.6 | 11 |
|  | IL-6/17/23i | 66 | 1.0 | 4.7 | 6.3 | 7.8 | 9.8 |
|  | JAKi | 28 | 0.71 | 4.0 | 5.9 | 6.9 | 8.4 |
|  | TNFi mono | 166 | 0.45 | 3.6 | 5.3 | 7.1 | 9.1 |
|  | TNFi combi | 66 | 0.24 | 1.9 | 3.9 | 5.7 | 8.4 |
|  | Abatacept | 13 | 0.14 | 2.4 | 3.3 | 5.2 | 7.5 |
|  | Rituximab | 18 | 0.080 | 0.13 | 0.91 | 5.6 | 8.5 |
| 2nd+24w | no_med | 71 | 0.65 | 3.1 | 4.9 | 6.5 | 9.2 |
|  | csDMARD | 45 | 1.1 | 3.6 | 5.4 | 7.3 | 10.3 |
|  | IL-6/17/23i | 63 | 0.43 | 2.7 | 4.3 | 6.0 | 8.8 |
|  | JAKi | 26 | 0.31 | 3.5 | 4.1 | 5.4 | 7.1 |
|  | TNFi mono | 160 | 0.25 | 1.3 | 2.4 | 4.2 | 9.3 |
|  | TNFi combi | 63 | 0.13 | 0.81 | 1.6 | 2.6 | 7.4 |
|  | Abatacept | 13 | 0.13 | 0.72 | 1.2 | 2.1 | 7.0 |
|  | Rituximab | 12 | 0.080 | 0.14 | 0.96 | 3.4 | 7.3 |
| BL = baseline (day of 1st vaccine dose, before vaccination), 2nd+4w/12w/24w = 4/12/24 weeks post 2nd vaccine dose. No med = currently on no medication; csDMARD = conventional synthetic disease-modifying antirheumatic drugs in mono or combination therapy with GC (glucocorticoids); IL-6/17/23i = interleukin 6/17/23 inhibitors in mono or combination therapy with csDMARD/csDMARD & GC; JAKi = janus kinase inhibitors in mono or combination therapy with csDMARD/csDMARD & GC; TNFi mono = tumor necrosis factor inhibitor as monotherapy, TNFi combi = TNFi in combination therapy with csDMARD/GC/csDMARD & GC; Abatacept in mono or combination therapy with csDMARD/csDMARD & GC; Rituximab in mono or combination therapy with csDMARD/csDMARD & GC. The following treatment groups with 5 or fewer participants are not shown here: GC monotherapy and PDE4i (phosphodiesterase-4 inhibitor) in mono or combination therapy with csDMARD. | | | | | | | |

**Table S4. Summary statistics for antibody levels by vaccine and SARS-CoV-2 infection status** for IRD patients (antibody levels reported in terms of optical density ratios).

| SARS-CoV-2 infection | Timepoint | Vaccine | Number of samples | Min. | 1^st^ quartile | Median | 3rd quartile | Max. |
| --- | --- | --- | --- | --- | --- | --- | --- | --- |
| SARS-CoV-2 naive | BL | mRNA-1273 | 232 | 0.050 | 0.080 | 0.10 | 0.13 | 1.6 |
|  |  | BNT162b2 | 275 | 0.050 | 0.080 | 0.10 | 0.13 | 0.66 |
|  | 2nd+4w | mRNA-1273 | 228 | 0.060 | 7.4 | 8.2 | 8.8 | 11 |
|  |  | BNT162b2 | 267 | 0.090 | 6.0 | 7.3 | 8.3 | 10 |
|  | 2nd+12w | mRNA-1273 | 224 | 0.090 | 5.1 | 7.0 | 8.0 | 11 |
|  |  | BNT162b2 | 262 | 0.080 | 3.1 | 5.0 | 6.4 | 9.9 |
|  | 2nd+24w | mRNA-1273 | 210 | 0.080 | 2.5 | 4.4 | 6.5 | 10.3 |
|  |  | BNT162b2 | 251 | 0.10 | 1.3 | 2.5 | 4.3 | 7.8 |
| SARS-CoV-2 recovered | BL | mRNA-1273 | 28 | 0.090 | 0.33 | 0.84 | 2.2 | 9.8 |
|  |  | BNT162b2 | 30 | 0.070 | 0.44 | 0.97 | 2.7 | 5.0 |
|  | 2nd+4w | mRNA-1273 | 27 | 6.6 | 8.5 | 9.1 | 10 | 11 |
|  |  | BNT162b2 | 30 | 4.6 | 8.1 | 8.6 | 9.3 | 10 |
|  | 2nd+12w | mRNA-1273 | 27 | 3.2 | 7.4 | 8.0 | 9.3 | 10.4 |
|  |  | BNT162b2 | 29 | 4.2 | 5.4 | 7.3 | 8.0 | 10.6 |
|  | 2nd+24w | mRNA-1273 | 25 | 1.3 | 5.4 | 7.1 | 8.7 | 10.3 |
|  |  | BNT162b2 | 27 | 1.9 | 4.3 | 5.8 | 7.2 | 10.3 |
| BL = baseline (day of 1st vaccine dose, before vaccination), 2nd+4w/12w/24w = 4/12/24 weeks post 2nd vaccine dose. | | | | | | | | |

**References**

1 Moor MB, Suter-Riniker F, Horn MP, *et al.* Humoral and cellular responses to mRNA vaccines against SARS-CoV-2 in patients with a history of CD20 B-cell-depleting therapy (RituxiVac): an investigator-initiated, single-centre, open-label study. *Lancet Rheumatol* 2021; **3**: e789–97.
